# Transthoracic M-mode Echocardiography Demonstrating Cardiac Tamponade

**DOI:** 10.7759/cureus.20106

**Published:** 2021-12-02

**Authors:** Christian C Toquica Gahona, Abi Watts, Keval V Patel

**Affiliations:** 1 Internal Medicine, Saint Peter’s University Hospital, New Brunswick, USA; 2 Internal Medicine, Saint Peter's University Hospital , New Brunswick, USA; 3 Cardiology, Rutgers-Robert Wood Johnson University Hospital, New Brunswick, USA; 4 Cardiology, Saint Peter’s University Hospital, New Brunswick, USA

**Keywords:** pericardial effusion, pericardiocentesis, m-mode, transthoracic echocardiogram, cardiac tamponade

## Abstract

A 67-year-old man presented to the emergency department for two weeks of progressive shortness of breath with orthopnea and new-onset back pain. On admission, vital signs were normal, and physical exam was remarkable for jugular vein distention with the rest of the cardiovascular exam in normal limits. A bedside transthoracic echocardiogram showed a large circumferential pericardial effusion with M-mode analysis revealed diastolic collapse of the right ventricle (RV) and > 40% tricuspid inflow respiratory variation in Doppler. Emergency pericardiocentesis yielded 800 cm3 of yellow-colored fluid. Subsequently, the patient underwent lymph node biopsy showing tumor cells consistent with squamous cell carcinoma of unknown origin. This case highlights the use of bedside echocardiogram and M-mode imaging for the diagnosis of cardiac tamponade.

## Introduction

Cardiac tamponade occurs when intrapericardial pressure impedes normal cardiac filling [1]. It is a medical emergency that, left untreated, can cause hemodynamical instability and lead to cardiac arrest. Although cardiac tamponade is a clinical diagnosis, physical findings have low diagnostic yield. The use of echocardiography plays a critical role in patients presenting with pericardial effusion (PE), allowing a quick assessment of the hemodynamic status and guidance of pericardiocentesis [2]. We present a case of an insidious onset of cardiac tamponade with negative clinical signs and describe the use of M-mode echocardiography in this setting.

## Case presentation

A 67-year-old man presented to the emergency department for severe back pain and two weeks of progressive shortness of breath at rest and orthopnea. On review of symptoms, he mentioned having one week of loss of appetite and unquantified weight loss. He did not have leg swelling, chest pain, cough, or fever. His past medical history includes hypertension, hyperlipidemia, coronary artery disease, previous stroke over one year ago, atrial fibrillation on apixaban, and right-sided renal cell cancer status post nephrectomy one year prior to presentation. The patient was a former smoker of one pack a day for over 40 years.

**Figure 1:**
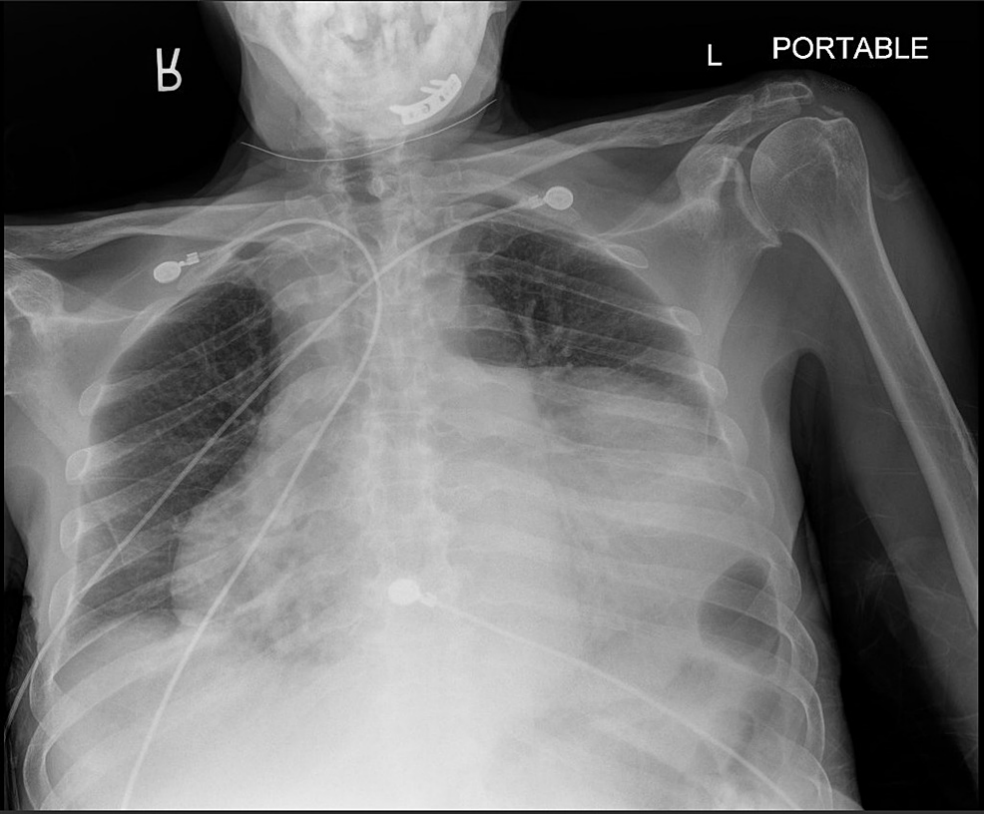
Anteroposterior X-ray of the chest Evidence of a very large pericardial effusion with a classic “water bottle shape” heart.

On admission, blood pressure was 117/64 mmHg, heart rate of 64 beats/min, respiratory rate of 16 breaths/minute, oxygen saturation was 99% breathing ambient air, temperature 98.2 degrees Fahrenheit, jugular vein distention was appreciated, cardiac sounds were normal with no rub, murmurs, or other extra sounds, there was no hepatomegaly or leg edema. Chest X-ray showed a boot-shaped cardiac silhouette concerning for pericardial effusion (Figure 1).

**Figure 2:**
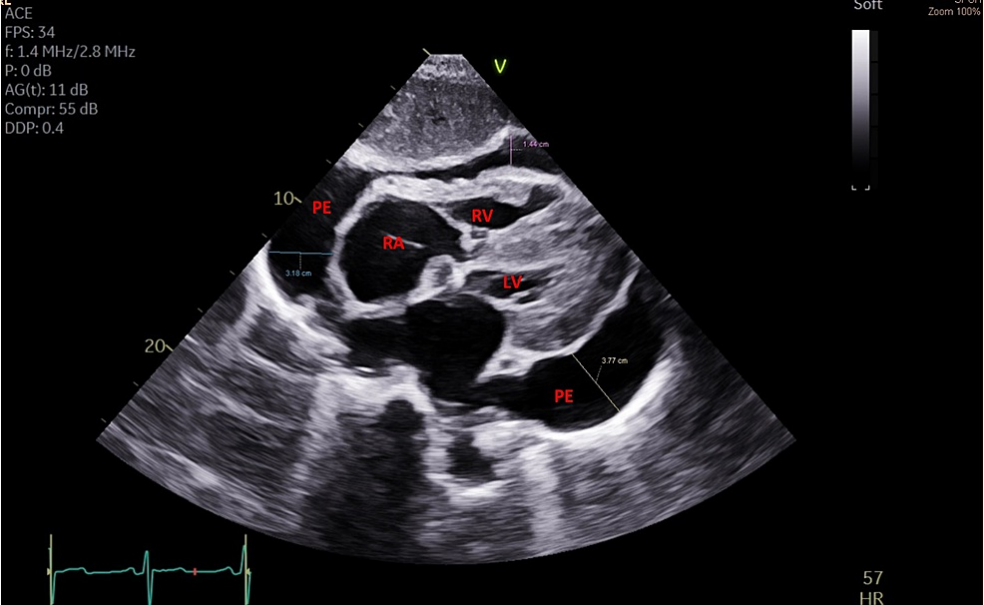
Transthoracic echocardiogram (TTE) subcostal view A large circumferential pericardial effusion with early diastolic right ventricle free-wall inversion. LV (Left ventricle), PE (Pericardial effusion), RA (Right atrium), RV (Right ventricle).

The electrocardiogram (ECG) showed sinus rhythm at 61 bpm, normal voltages with no electrical alternans. A bedside transthoracic echocardiogram (TTE) showed a large circumferential pericardial effusion (Figure 2).

**Figure 3:**
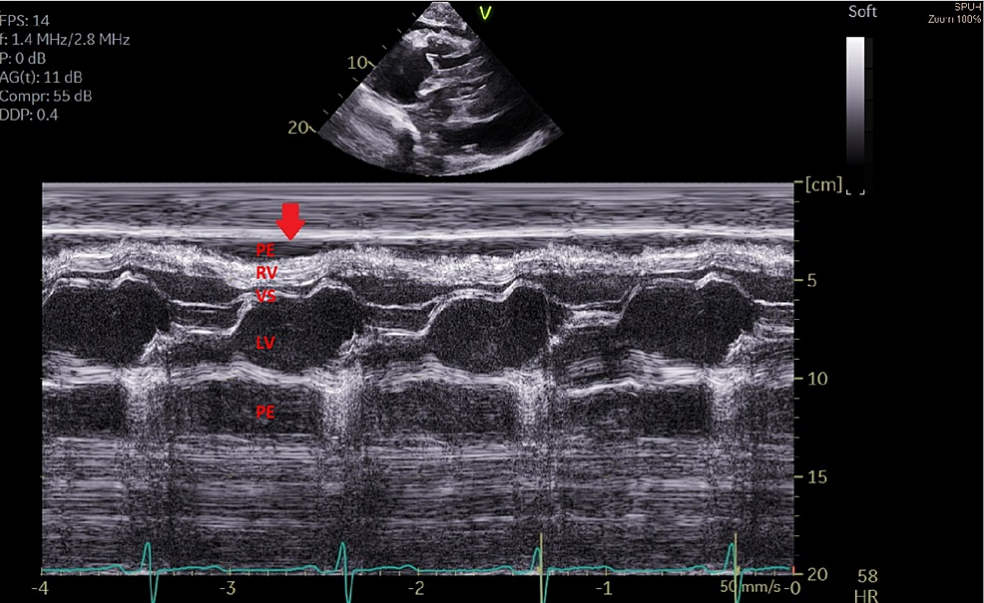
Transthoracic echocardiogram (TTE) M-mode Evidence of right ventricle collapse in early diastole (Red arrow). LV (Left ventricle), PE (Pericardial effusion), RA (Right atrium), RV (Right ventricle),

M-mode analysis revealed right atrial (RA) collapse >30% (Figure 3), and diastolic collapse of the right ventricle (RV). 

**Figure 4:**
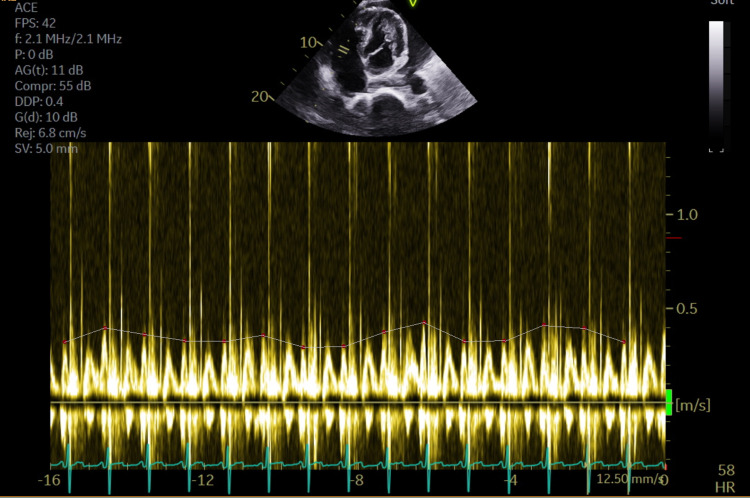
Transthoracic echocardiogram (TTE) doppler Images showing > 40% tricuspid inflow respiratory variation. The grey line has been drawn for visual appreciation.

Doppler imaging showed > 40% tricuspid inflow respiratory variation (Figure 4). The right ventricular systolic pressure was estimated at 44 mmHg, which is mildly elevated. The inferior vena cava (IVC) was normal in size, with less than 50% reduction in diameter of dilated vena cava during inspiration. The left ventricle had concentric hypertrophy with normal function (ejection fraction 60%).

Emergency pericardiocentesis yielded 800 cm3 of yellow-colored fluid. Repeated immediate TTE showed trace pericardial fluid, and a control TTE on day 5 post-procedure showed no re-accumulation of the fluid. A complete analysis of the fluid was noticeable for reactive mesothelial cells, histiocytes and negative for microorganisms and malignancy. A computerized tomography (CT scan) of the abdomen and pelvis was performed given the history of malignancy and the patient’s new-onset back pain which found multiple enlarged lymphadenopathies within the mediastinum, retroperitoneal, and mesenteric lymphadenopathy, with no bone metastasis or fractures. The patient underwent a CT-guided biopsy of retroperitoneal lymph nodes with cytology showing tumor cells positive for CK7, PAN-CYTOKERATIN, P63, P40 and negative for CK20, TTF-1, RCC, PAX8 consistent with squamous cell carcinoma of unknown origin.

The patient was medically cleared and was discharged from the hospital and had a favorable clinical course without recurrence of the PE after one month follow-up.

## Discussion

Cardiac tamponade is a medical emergency that requires a high degree of clinical suspicion for its recognition and prompt treatment. The clinical signs of cardiac tamponade were described by Dr. Claude Beck in 1935 initially as two triads of signs in patients with pericardial tamponade: 1) hypotension, venous distension, and diminished heart sounds in acute tamponade, and 2) high venous pressure, ascites, and diminished heart sounds in chronic cardiac compression [3]. However, in clinical practice, jugular venous distension, hypotension, and diminished heart sounds are found in around 54%, 28%, and 22% of the cases, respectively [4].

Symptoms such as dyspnea, chest discomfort, peripheral edema, fatigability are caused by increased filling pressures and limited cardiac output. It is interesting that, despite the degree of effusion and evidence of tamponade in imaging studies, symptoms can be mild in subacute pericardial effusions as described in the present case. In this clinical scenario, the use of chest X-ray, ECG, and echocardiography help in identifying cardiac tamponade [4].

The current etiology of pericardial effusions has shifted from infectious causes to malignancy and iatrogenic causes from cardiac interventions [5]. Other conditions such as hypothyroidism, autoimmune conditions, malignancy, tuberculosis, and myocardial infarction must also be ruled out.

Echocardiography is the diagnostic modality of choice for cardiac tamponade. The presence of right ventricle inversion exaggerated respiratory variation in peak mitral (>30%) and tricuspid (>60%) blood flow velocities in doppler, and dilated IVC with absent respiratory variation (IVC plethora) indicate impending tamponade [6-7]. Two of the three indicators use M-mode imaging.

Emergent pericardiocentesis is the treatment of choice for cardiac tamponade, and physicians must be aware that small increments of fluid in the pericardial sac may produce critical cardiac compression and lead to shock (last-drop phenomenon) [8]. In subacute and chronic effusions, there is a high chance of recurrence and should be followed by echocardiograms in 24 hours, and as much as 20% of patients will need pericardiectomy [1].

## Conclusions

History and clinical examination have limited performance in diagnosing cardiac tamponade. The use of echocardiographic features to support the hemodynamical compromise in patients with pericardial effusion is highly encouraged. Echocardiography is a useful tool to support emergent treatment decisions in patients with cardiac tamponade.
